# Defocus correction and noise reduction using a hybrid ptychography and Centre‐of‐Mass algorithm

**DOI:** 10.1111/jmi.70010

**Published:** 2025-08-21

**Authors:** Zhiyuan Ding, Chen Huang, Adrián Pedrazo‐Tardajos, Angus I Kirkland, Peter D Nellist

**Affiliations:** ^1^ Department of Materials University of Oxford Oxford UK; ^2^ The Rosalind Franklin Institute Harwell Science and Innovation Campus Didcot UK; ^3^ Electron Physical Sciences Imaging Centre Diamond Light Source Ltd. Harwell Science and Innovation Campus Didcot UK

**Keywords:** 4DSTEM, aberration correction, Centre‐of‐Mass, ptychography, TEM

## Abstract

Integrated Centre‐of‐Mass (iCOM) is a widely used phase‐contrast imaging method based on Centre‐of‐Mass (COM), which makes use of a 4D Scanning Transmission Electron Microscopy (STEM) dataset using an in‐focus probe. In this paper, we introduce a novel approach that combines Single‐Side Band (SSB) ptychography with COM and iCOM, termed Side Band masked Centre‐of‐Mass (SBm‐COM) and integrated Centre‐of‐Mass (SBm‐iCOM) which is applicable to weak‐phase objects. This method compensates for residual aberrations in 4DSTEM datasets while also reducing the noise contribution up to the 2α resolution limit. The aberration compensation and noise filtering features make the SBm‐(i)COM suitable for samples that are difficult to focus or those that require minimal electron fluence. SBm‐iCOM transfers the same information as SSB ptychography but results in an intrinsic transfer function that enhances low‐frequency information.

## LAY DESCRIPTION

Electric fields in materials will deflect an electron beam transmitted through the sample. In a scanning transmission electron microscope (STEM), the image is formed by detecting scattering from a focused beam of electrons that is scanned in a raster pattern over the sample. For each probe position, a diffraction pattern is formed by the transmitted electrons and collection of these diffraction patterns for each probe position forms a so‐called 4DSTEM data set. It has been previously been shown that detecting the intensity centre of mass in each diffraction pattern and plotting its displacement as a function of illuminating probe position forms an image of the electric fields in the sample. This technique requires a well‐focused beam, however. An alternative approach to processing the 4DSTEM data set is to use a method referred to as ptychography, which allows any lens aberrations, including defocus, to be measured and corrected. Here we combine ptychography with centre of mass imaging to create a hybrid imaging mode that allows for field mapping using defocused or otherwise aberrated STEM probes.

## 1 INTRODUCTION

Centre‐of‐Mass (COM) is a phase‐contrast imaging method in Scanning Transmission Electron Microscopy (STEM). As an electron beam scans the sample, internal electric or magnetic fields of the sample can deflect the beam, causing a shift in the centre of mass of the convergent beam electron diffraction (CBED) pattern on the diffraction plane. The contrast in COM and integrated COM (iCOM) arises from this shift. By analysing the shift in the centre of mass of the CBED pattern, information about the internal electric or magnetic fields within the sample can be reconstructed. Related scanning phase‐contrast methods have been previously reported^1^, ^2^ including a prototype of Centre‐of‐Mass (COM).^3^ Subsequently, differential phase‐contrast (DPC) methods, which utilise segmented detectors, have been reported^4^, ^5^ and can be considered an approximation of COM methods for pixelated detectors. Depending on post processing, COM can be further divided into COMx/y (Centre‐of‐Mass shift in specific directions) and iCOM (integrated Centre‐of‐Mass).^4^ The linearity of DPC/COM contrast has also been discussed,^6^ showing that for a phase object, the contrast of COMx/y and iCOM images are proportional to the projected electric field and potential distribution respectively.^4^ With the development of 4DSTEM^7^ techniques, COM/iCOM and related DPC methods are now frequently applied to 4DSTEM datasets to obtain fast (or real‐time) phase‐contrast images^8^ which have been used to map projected electric fields and potential distributions.^9^, ^10^, ^11^, ^12^

In principle, the COM/iCOM method requires an in‐focus probe to achieve high resolution as a defocused probe will blur COM/iCOM images due to a convolution between the probe function and the transmission (object) function.^4^, ^11^This sets a limit when minimising the fluence to control beam damage of radiation sensitive samples, as fine‐tuning the in‐focus probe leads to additional fluence on the sample which does not contribute signal but causes damage.

In this paper, we propose an adaptation of Centre‐of‐Mass methods for use with defocused probes, by Single‐Side Band (SSB) ptychography^13^ and Centre‐of‐Mass denoted as Side Band masked COM (SBm‐COM) and the integrated version SBm‐iCOM. SBm‐(i)COM is based on the Weak Phase Object Approximation (WPOA) as is SSB ptychography and can correct aberrations (including defocus) and reduce noise after 4DSTEM data collection. In this method, a Fourier transform with respect to the probe scanning positions of the 4DSTEM dataset, that is, 4D array G, is calculated and a mask similar to that used in SSB ptychography is applied to G. This mask optimally filters the original dataset, removing pixels with no phase‐contrast information containing only noise and correcting phase variations due to aberrations.^14^ As such, in addition to aberration correction, this method effectively reduces the high‐frequency noise present in traditional iCOM images.

## 2 METHOD

A sample transmission function ψ(R) under the Weak Phase Object Approximation (WPOA) is considered. A probe function P(R), scans the sample, where the scanning coordinate $R_{p}$ indicates the probe position during the scan. Thus, the exit wave function $EW(R,R_{p})$ can be expressed as:

(1) $$EW\left(R,R_{p}\right)=P\left(R-R_{p}\right)\cdot \psi \left(R\right).$$

This exit wave is propagated to the diffraction plane by a Fourier transform with respect to R and the wave function at the diffraction plane is denoted as $M(K_{f},R_{p})$. Furthermore, $M(K_{f},R_{p})$ can be represented by a result of a convolution:

(2) $$\begin{array}{ccc} M\left(K_{f},R_{p}\right) & = & F\left\{\mathrm{EW}\left(R,R_{p}\right)\right\}\\ & = & \int P\left(R-R_{p}\right)\cdot \psi \left(R\right)\cdot \exp\left(-2\pi iR\cdot K_{f}\right)dR\\ & = & \left[A\left(K_{f}\right)\exp\left(-2\pi iK_{f}\cdot R_{p}\right)\right]{⊗}_{K_{f}}\Psi \left(K_{f}\right), \end{array}$$

where ${⊗}_{K_{f}}$ represents the convolution operation with respect to $K_{f}$. Based on Equation (2), the intensity recorded in a 4DSTEM dataset is given by ${|M(K_{f},R_{p})|}^{2}$.

For the traditional COM/iCOM algorithm, $COM_{x}\left(R_{p}\right)$, $COM_{y}\left(R_{p}\right)$ and $\mathrm{COM}(R_{p})$ are calculated as:

(3) $$\begin{array}{c} \mathrm{CO}M_{x}\left(R_{p}\right)=\int K_{\mathrm{fx}}\cdot {\left|M\left(K_{f},R_{p}\right)\right|}^{2}dK_{f}\\ \mathrm{CO}M_{y}\left(R_{p}\right)=\int K_{\mathrm{fy}}\cdot {\left|M\left(K_{f},R_{p}\right)\right|}^{2}dK_{f}\\ \mathrm{COM}\left(R_{p}\right)=\left(\mathrm{CO}M_{x}\left(R_{p}\right),\mathrm{CO}M_{y}\left(R_{p}\right)\right) \end{array}$$

and subsequently, $iCOM(R_{p})$ is calculated from $\mathrm{COM}(R_{p})$ ^4^ as:

(4) $$iCOM\left(R_{p}\right)=F^{-1}\left\{\frac{Q_{p}\cdot F\left\{\mathrm{COM}\left(R_{p}\right)\right\}}{2\pi iQ_{p}^{2}}\right\},$$

where F and $F^{-1}$ are Fourier and inverse Fourier transform operators.

In ptychography, ${|M(K_{f},R_{p})|}^{2}$ can be Fourier transformed with respect to $R_{p}$, as in Equation (5), with the resulting Fourier transform represented as $G(K_{f},Q_{p})$.^13^, ^15^

(5) $$G\left(K_{f},Q_{p}\right)=F_{R_{p}}\left\{{\left|M\left(K_{f},R_{p}\right)\right|}^{2}\right\}.$$

For comparison in SBm‐COM, a Fourier transform is applied to $COM_{x}\left(R_{p}\right)$ and $COM_{y}\left(R_{p}\right)$. Since the variable $R_{p}$ is independent with respect to an integration variable $K_{f}$,  the order of the Fourier transform and the integral calculation can be interchanged. Therefore, a Fourier transform of $COM_{x}\left(R_{p}\right)$ and $COM_{y}\left(R_{p}\right)$, denoted as $com_{x}\left(Q_{p}\right)$ and $com_{y}\left(Q_{p}\right)$, can be calculated using Equation (6). The function $G(K_{f},Q_{p})$ is the Fourier transform of ${|M(K_{f},R_{p})|}^{2}$ with respect to $R_{p}$, defined by Equation (5).

(6) $$\begin{array}{ccc} \mathrm{co}m_{x}\left(Q_{p}\right) & = & F_{R_{p}}\left\{\mathrm{CO}M_{x}\left(R_{p}\right)\right\}\\ & = & F_{R_{p}}\left\{\int K_{\mathrm{fx}}\cdot {\left|M\left(K_{f},R_{p}\right)\right|}^{2}dK_{f}\right\}\\ & = & \int K_{\mathrm{fx}}G\left(K_{f},Q_{p}\right)dK_{f}\\ \mathrm{co}m_{y}\left(Q_{p}\right) & = & F_{R_{p}}\left\{\mathrm{CO}M_{y}\left(R_{p}\right)\right\}\\ & = & F_{R_{p}}\left\{\int K_{\mathrm{fy}}\cdot {\left|M\left(K_{f},R_{p}\right)\right|}^{2}dK_{f}\right\}\\ & = & \int K_{\mathrm{fy}}G\left(K_{f},Q_{p}\right)dK_{f}. \end{array}$$

Furthermore, $G(K_{f},Q_{p})$ can be rewritten as a convolution, as in Equation (7).^16^, ^17^

(7) $$\begin{array}{ccc} G\left(K_{f},Q_{p}\right) & = & F_{R_{p}}\left\{{\left|M\left(K_{f},R_{p}\right)\right|}^{2}\right\}\\ & = & A\left(K_{f}\right)A^{*}\left(K_{f}+Q_{p}\right){⊗}_{K_{f}}\Psi \left(K_{f}\right)\Psi^{*}\\ & & \left(K_{f}-Q_{p}\right). \end{array}$$

The Weak Phase Object Approximation (WPOA) can be incorporated into Equation (7) by assuming that $\psi_{s}$ is a purely imaginary function used to represent a transmission function that satisfies the WPOA condition, and $\Psi_{s}$ is its Fourier transform. The relationship between the original transmission function ψ and $\;\psi_{s}$ is:

(8) $$\psi \left(R_{p}\right)\approx 1+\psi_{s}\left(R_{p}\right).$$

Under the WPOA, Equation (7) can be rewritten by replacing Ψ with $\Psi_{s}$ and $G(K_{f},Q_{p})$ can be further simplified^13^ as in Equation (9).

(9) $$\begin{array}{ccc} & & G\left(K_{f},Q_{p}\right)\approx {\left|A\left(K_{f}\right)\right|}^{2}\delta \left(Q_{p}\right)+A\left(K_{f}\right)A^{*}\left(K_{f}+Q_{p}\right)\\ & & \Psi_{s}^{*}\left(-Q_{p}\right)+A^{*}\left(K_{f}\right)A\left(K_{f}-Q_{p}\right)\Psi_{s}\left(Q_{p}\right), \end{array}$$

where $\Psi_{s}\left(Q_{p}\right)$ represents the Fourier transform of $\psi_{s}\left(R_{p}\right)$. In Equation (9), the first item is 0 when $Q_{p}\neq 0$, and has no influence on nonzero spatial frequencies. Alternatively, the first item can be considered as a constant background which does not affect the contrast of the reconstruction.

**FIGURE 1:**
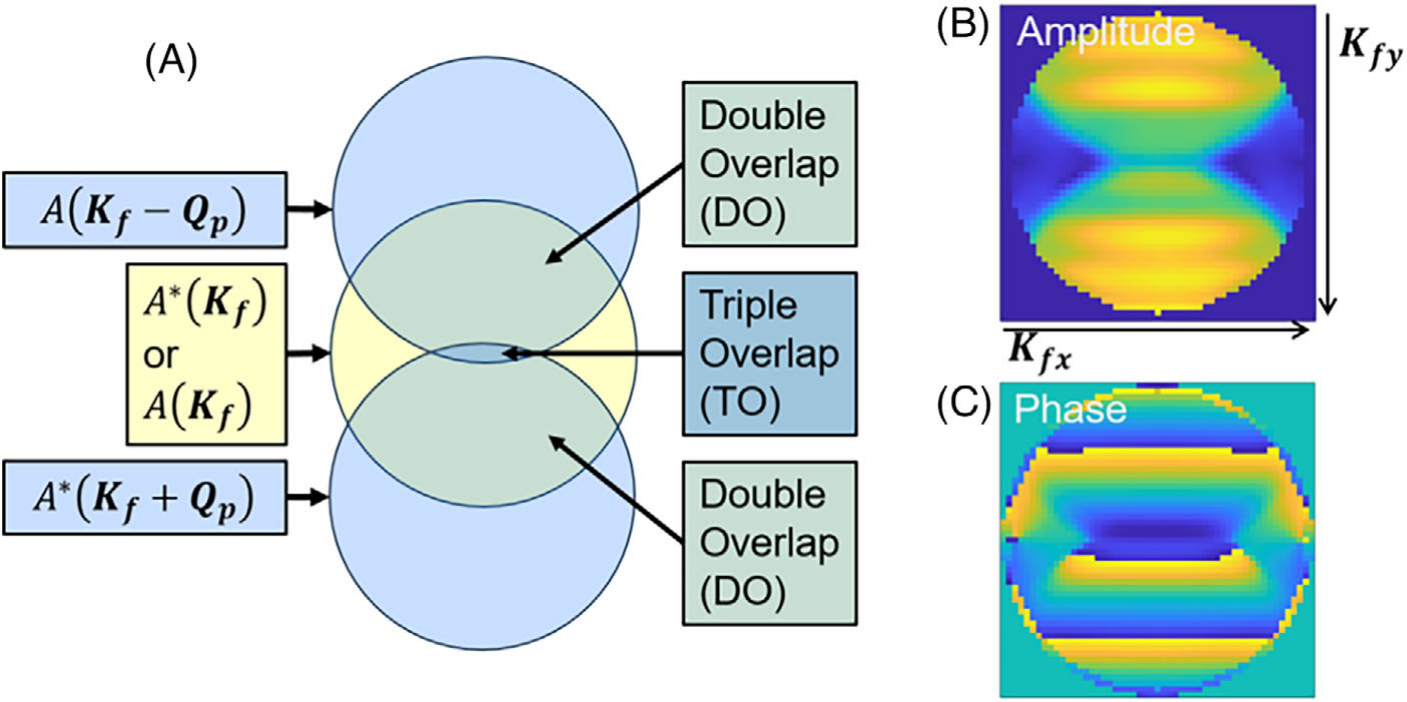
(A) Schematic showing the G matrix and DO/TO regions. (B, C) slices along G from the DO region. The structures caused by defocus can be seen in both the amplitude (B) and phase (C) of $G(K_{f},Q_{p})$. Acceleration voltage: 80 kV, defocus 10 nm.

The second and third items are those used in SSB ptychography reconstruction.^13^ For a specific $Q_{p}$, $A\left(K_{f}\right)A^{*}\left(K_{f}+Q_{p}\right)$ or $A^{*}\left(K_{f}\right)A\left(K_{f}-Q_{p}\right)$ represents the overlapping region of an aperture function, $A(K_{f})$ or $A^{*}\left(K_{f}\right)$ (or the direct beam), shifted by $Q_{p}$ or $-Q_{p}$ in the $K_{f}$ plane, as shown in Figure 1A. The region overlapped only by the zero‐shift aperture function and a beam at $-Q_{p}$ or $Q_{p}$ is denoted as a ‘double‐overlap (DO) region’. The region overlapped by $A(K_{f})$ or $A^{*}\left(K_{f}\right)$ and both $-Q_{p}$ and $Q_{p}$ beams is denoted as the ‘triple‐overlap (TO) region’. A schematic diagram showing the DO and TO regions is shown in Figure 1A. The aperture function $A(K_{f})$ is modelled as a top‐hat function at $|K_{f}|\leq \alpha$, where α is the convergence angle, with a phase modified by the objective lens axial aberrations. The phase information in $\Psi_{s}\left(Q_{p}\right)$ can be easily analysed in the DO regions, where only one diffracted beam interferes with the nonshifted aperture function. An example of a simulated $G(K_{f},Q_{p})$ slice with defocus included is shown in Figure 1B and C.

In Figure 1A, the highest $|Q_{p}|$ value for which $G(K_{f},Q_{p})$ in Equation (9) remains nonzero is at $|Q_{p}|=2\alpha$. Here we have expressed $Q_{p}$ in terms of a scattering angle but it could equivalent be expressed as a reciprocal distance. When $|Q_{p}|>2\alpha$, $A(K_{f})$ and $A(K_{f}+Q_{p})$ have no overlapping areas, resulting in $G(K_{f},Q_{p})=0$.

Equation (9) shows that sample information ($\Psi_{s}$) is only contained in the two DO regions. Outside the DO region, the terms $A\left(K_{f}\right)A^{*}\left(K_{f}+Q_{p}\right)$ and $A^{*}\left(K_{f}\right)A\left(K_{f}-Q_{p}\right)$ are zero, blocking information transfer. The TO region is zero if the 4DSTEM dataset is in‐focus, or takes values of a combination of $\Psi_{s}$ and $\Psi_{s}^{*}$ when out of focus, from which it is difficult to separate information in $\Psi_{s}$.^13^ Importantly, Equation (9) provides a simple solution for $\Psi_{s}$ or $\Psi_{s}^{*}$ by introducing masks for the DO regions, allowing them to be simplified into 2 separate terms that contain object information.

The information within the DO area can also be used to estimate aberration parameters in the 4DSTEM dataset.^18^ As shown in Figure 1C, for a specific $Q_{p}$, the phase distribution on $G(K_{f},Q_{p})$, with respect to the $K_{f}$ axes, is determined only by either $A\left(K_{f}\right)A^{*}\left(K_{f}+Q_{p}\right)$ or $A^{*}\left(K_{f}\right)A\left(K_{f}-Q_{p}\right)$. Therefore, by applying a Singular Value Decomposition (SVD) algorithm, the aberration parameters that define the aperture $A(K_{f})$ function can be extracted. The complete algorithm is detailed in Ref. (18).

Post reconstruction aberration correction can be achieved by modifying the DO mask. The relevant mask is generated by multiplying $A^{*}\left(K_{f}\right)A\left(K_{f}+Q_{p}\right)$ and $A\left(K_{f}\right)A^{*}\left(K_{f}-Q_{p}\right)$, with the DO term in $G(K_{f},Q_{p})$. This mask function, $m(K_{f},Q_{p})$, including aberrations can be defined as:

(10) $$m\left(K_{f},Q_{p}\right)=\left\{\begin{array}{c} A^{*}\left(K_{f}\right)A\left(K_{f}+Q_{p}\right),\;\;\;\;\;\;\;\;\;\;\;\;\;\mathrm{in}\;Q_{p}\;\mathrm{beam}\;\mathrm{DO}\;\mathrm{region}\;\\ A\left(K_{f}\right)A^{*}\left(K_{f}-Q_{p}\right),\;\;\;\;\;\;\;\;\;\;\mathrm{in}-Q_{p}\;\mathrm{beam}\;\mathrm{DO}\;\mathrm{region}\\ 0,\;\;\;\;\;\;\;\;\;\;\;\;\;\;\;\;\;\;\;\;\;\;\;\;\;\;\;\;\;\;\;\;\;\;\;\mathrm{out}\;\mathrm{of}\;\mathrm{DO}\;\mathrm{region}.\; \end{array}\right.$$

This mask for DO regions is applied in the SBm‐COM, to $G(K_{f},Q_{p})$ in Equation (6). The traditional COM method can be regarded as having a mask function $m(K_{f},Q_{p})=1$ at the same position in Equation (6). The corresponding masked expressions for $com_{x}\left(Q_{p}\right)$ and $com_{x}\left(Q_{p}\right)$ are denoted as $com_{x}^{f}\left(Q_{p}\right)$ and $com_{y}^{f}\left(Q_{p}\right)$, respectively, where the superscript ‘f’indicates ‘filtered’, referring to the filtering of pixels outside the DO region.

(11) $$\begin{array}{c} \;\mathrm{co}m_{x}^{f}\left(Q_{p}\right)=\int K_{\mathrm{fx}}G\left(K_{f},Q_{p}\right)m\left(K_{f},Q_{p}\right)dK_{f}\\ \mathrm{co}m_{y}^{f}\left(Q_{p}\right)=\int K_{\mathrm{fy}}G\left(K_{f},Q_{p}\right)m\left(K_{f},Q_{p}\right)dK_{f} \end{array}.$$

When the mask is used in SBm‐COM methods, both DO regions are included in the integrals in Equation (11). This differs from traditional SSB ptychography, where the two DO regions are summed separately. The reason for this is that the two DO regions are complex conjugates (but contain identical information) and if they are summed together in SSB their phase components cancel. On the other hand, since the two DO regions are always symmetric with respect to the origin, the sign of $K_{fx}$ or $K_{fy}$ is always opposite for the two regions. Therefore, when multiplied by $K_{fx}$ or $K_{fy}$ in the integrals in Equation (11), their phases do not cancel. Consequently, they can be integrated together. However, the two DO regions contains identical information (including noise), so using two regions does not imply more information recovery than in SSB ptychography.

In $com_{x}^{f}\left(Q_{p}\right)$ and $com_{y}^{f}\left(Q_{p}\right)$, there is an intrinsic filtering effect based on different $Q_{p}$ values as the area of mask varies when $Q_{p}$ is changed. This acts as a bandpass filter for the noise but leaves the signal unchanged, suppressing high‐frequency noise near the limit at 2α. The noise due to electron counting statistics is close to a uniform distribution across the whole of the central disc whereas the algorithm only uses the parts of the disc where the signal is above the noise leading to noise rejection. This effect is strongest at low frequencies and at frequencies close to 2α where in both cases the DO area is relatively small. This filtering is also incorporated into the contrast transfer function of SBm‐COM, as discussed later.

After applying the mask, filtered COM images can be calculated by performing an inverse Fourier transform on $com_{x}^{f}\left(Q_{p}\right)$ and $com_{y}^{f}\left(Q_{p}\right)$ as:

(12) $$\begin{array}{c} COM_{x}^{f}\left(R_{p}\right)=F^{-1}\left\{com_{x}^{f}\left(Q_{p}\right)\right\}\\ COM_{y}^{f}\left(R_{p}\right)=F^{-1}\left\{com_{y}^{f}\left(Q_{p}\right)\right\}\\ \mathrm{CO}M^{f}\left(R_{p}\right)=\left(COM_{x}^{f}\left(R_{p}\right),COM_{y}^{f}\left(R_{p}\right)\right) \end{array}.$$

Finally, filtered $iCOM^{f}\left(R_{p}\right)$ can be calculated using the traditional algorithm with a replacement of $\mathrm{co}m^{f}\left(Q_{p}\right)$ as:

(13) $$iCOM^{f}\left(R_{p}\right)=F^{-1}\left\{\frac{Q_{p}\cdot \mathrm{co}m^{f}\left(Q_{p}\right)}{2\pi iQ_{p}^{2}}\right\}.$$

Importantly, all of the filtered $\mathrm{CO}M^{f}$, $iCOM^{f}$, $dCOM^{f}$ are corrected for residual aberrations.

## 3 EXPERIMENTS

### 3.1 Sample preparation

A graphene sample was prepared by transferring graphene to a TEM grid using cellulose acetate butyrate (CAB).^19^, ^20^ The cellulose‐based polymer was prepared by mixing CAB (Merck) with ethyl acetate. The polymer solution was then deposited on top of CVD graphene grown on copper (Grolltex, USA) using a spin coater (Ossila Spin Coater). Subsequently, the copper foil was floated on an aqueous solution of ammonium persulphate (3.2 g dissolved in 100 mL of ultrapure water) until the copper foil was completely etched. After etching, the solution was replaced with ultrapure water by sequentially removing the etching solvent and adding fresh water. TEM grids (Au‐Flat 300 Mesh EMS) were introduced into the water solution on a filter paper placed at the bottom of a liquid container. Water was carefully removed using a pipette until the CAB+graphene layer was deposited on top of the grids. Finally, the graphene TEM grid was placed on activated carbon,^21^ heated to 310°C and left overnight to remove the polymer layer.

### 3.2 Data collection and reconstruction

4DSTEM datasets at different defoci were recorded at 80 kV, using a JEOL ARM300F microscope with a MerlinEM detector from Quantum Detectors. The convergence angle was 24.8 mrad and the scanning step was 0.022 nm. The experimental fluence for each 4DSTEM dataset was 3.19×10^6^ e^−^/nm^2^ and 4DSTEM focal series were recorded at defoci of 0, –7.5 and –22 nm.

**FIGURE 2:**
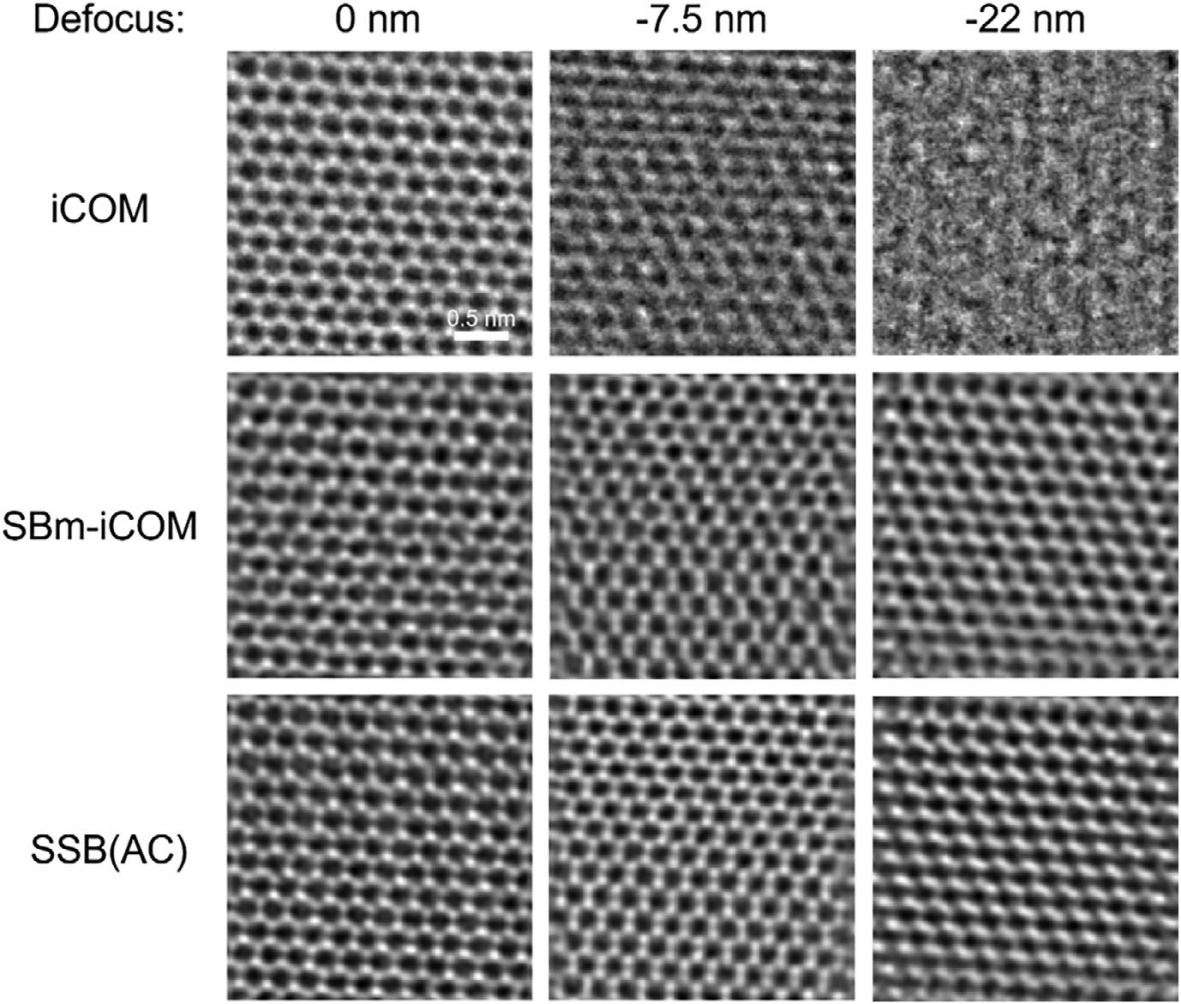
iCOM, SBm‐iCOM and SSB(AC) reconstructions for monolayer graphene with defoci of 0, –7.5 and –22 nm. SSB with aberration correction and SBm‐iCOM compensates defocus in the reconstructions for defoci of –7.5 and –22 nm. Acceleration voltage: 80 kV, convergence angle: 24.8 mrad, pixel size: 0.022 nm.

**FIGURE 3:**
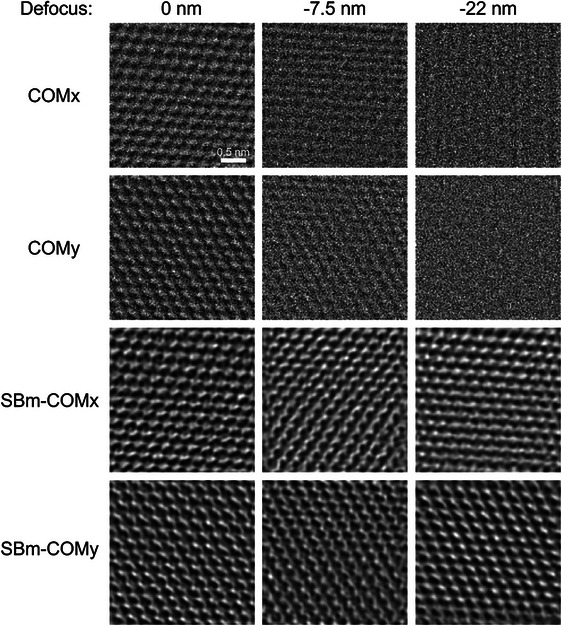
COMx/y and SBm‐COMx/y reconstructions for monolayer graphene. SBm‐COMx/y compensates defocus for defoci of –7.5 and –22 nm. Acceleration voltage: 80 kV, convergence angle: 24.8 mrad, pixel size: 0.022 nm. Defocus: 0, –7.5, –22 nm.

The focal series datasets were collected from the same sample area and reconstructed separately using COM/iCOM, SBm‐COM/iCOM and SSB (with aberration correction) algorithms. The defocus was changed in each dataset by changing the current in the objective lens and recording the same sample area for each defocus setting. Reconstructions are shown in Figure 2 (iCOM, SBm‐iCOM and SSB ptychography) and Figure 3 (COMx/y and SBm‐COMx/y). In the reconstruction, residual aberrations (other than defocus, shown in Table 1) were measured based on the dataset recorded at –7.5 nm defocus,^18^ and used in the reconstructions of datasets with –7.5 nm and –22 nm defoci. As discussed by Lazić et al.,^4^ experimental iCOM images can amplify low‐frequency noise. Hence, to avoid amplified low frequencies, a high‐pass filter (hard threshold) was applied to the iCOM images in Figure 2.

**TABLE 1:** Residual aberrations measured as described in the text.

$C_{1}$	−7.51 nm
$C_{12}-x,y$	1.13, 3.74 nm
$C_{21}-x,y$	−212, –64.4 nm
$C_{23}-x,y$	58.8, –101 nm
$C_{3}$	66 nm
$C_{32}-x,y$	−3.96, 1.20 µm
$C_{34}-x,y$	7.26, 0.826 µm

In Figures 2 and 3, with increasing defocus, at –7.5 and –22 nm, SBm‐COMx/y and SBm‐iCOM improve the reconstruction compared to COMx/y and iCOM. iCOM, in Figure 2, at a defocus of –22 nm, the reconstruction shows no lattice information, whereas at the same defocus SBm‐COM shows atomic resolution.

As shown in Figure 3, uncorrected COMx/y contains no useful information about the sample at a defocus of –22 nm. By comparison, SBm‐COMx/y contains lattice information at the same defocus. Furthermore, the high‐frequency noise in the in‐focus COMx/y reconstruction is reduced using SBm‐COMx/y.

This demonstrates that the SBm‐COMx/y and SBm‐iCOM algorithms can correct defocus post‐acquisition. Theoretically, the same algorithm can also be used to correct other low order aberrations. However, for experimental datasets, correcting aberrations other than defocus depends on accurate aberration measurements, which need to be independently measured.^18^, ^22^ The effectiveness of indirect aberration correction is limited by the precision of the aberration values provided to the SBm‐COM algorithm which can be measured from the phase variation in the DO region.^18^

As mentioned in the Methods section, the aberration estimation relies on measuring the phase distribution in $G(K_{f},Q_{p})$ in the double overlap region. For higher aberration levels, where the phase variation increases, the sampling on the $K_{f}$ plane within the DO area becomes a critical factor. Inadequate sampling can hinder accurate phase unwrapping, making aberration estimation more inaccurate. If the sampling is insufficient for reliable phase unwrapping, the resulting aberration estimation becomes unreliable.

Another common approach to obtain high‐order aberration parameters is to read them directly from the aberration corrector, if the microscope is equipped with one. However, the accuracy of these parameters depends on both the hardware and software of the corrector system. Moreover, aberration correctors can be unstable, and the actual aberration parameters may drift over a day. As a result, time gap between reading the parameters (typically after alignment) and data acquisition may introduce additional inaccuracies.

In conclusion, regardless of how the aberration parameters are calibrated, this aberration correction feature makes the SBm‐COMx/y and SBm‐iCOM algorithms adaptable to various imaging conditions, which is important for samples that are difficult to focus or those that require minimal electron fluence.

## 4 SIMULATIONS AND DISCUSSION

### 4.1 SBm‐iCOM for different fluences

In addition to aberration correction, SBm‐COM/iCOM offers another advantage over COM/iCOM as the SSB mask filters data outside the DO area that introduces noise without providing useful signal. Theoretically, if the sample can be regarded as a weak phase object and an aberration‐free probe is assumed, all phase‐contrast information is included in the SSB mask as shown in Equation (9).^23^ As a result, for SBm‐COM, filtering the area outside the SSB mask improves the signal‐to‐noise ratio of the data used for reconstruction. As $|Q_{p}|$ decreases to zero or increases to 2α from a frequency at α, the SSB mask area decreases and eventually reaches zero at a frequency corresponding to zero at 2α, inherently suppressing high‐frequency noise.

The denoise process of SBm‐iCOM is based on the physical principles of 4DSTEM, rather than relying on statistical noise‐smoothing techniques such as Principal Component Analysis (PCA).^24^, ^25^, ^26^ The mask $m(K_{f},Q_{p})$ selectively contains areas directly related to either $\Psi_{s}\left(Q_{p}\right)$ or $\Psi_{s}^{*}\left(-Q_{p}\right)$ in Equation (9), where the information of sample resides within the 4D array $G(K_{f},Q_{p})$, under the WPOA condition.

**FIGURE 4:**
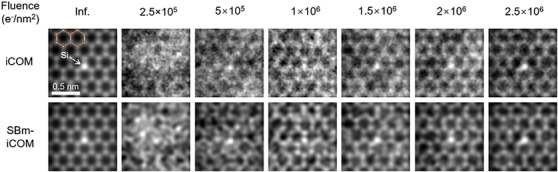
Reconstructions of simulated silicon‐replaced monolayer graphene for different fluences as shown. SBm‐iCOM suppresses high‐frequency noise under low fluences compared to iCOM. Voltage: 80 kV. Convergence angle: 22.5 mrad. Pixel size: 0.02 nm.

To demonstrate this denoising effect, Figure 4 shows a series of in‐focus simulations at different electron fluences using iCOM and SBm‐iCOM for the same sample (monolayer graphene with a silicon atom replacement). Compared to traditional iCOM, SBm‐iCOM reconstructs smoother images, particularly at fluences ranging from 5×10^5^ to 2.5×10^6^ e^−^/nm^2^, due to this intrinsic filtering effect.

### 4.2 Frequency transfer

We assume a sample that is a weak phase object for analysis of iCOM and SBm‐iCOM as in Equation (8). Under this approximation, $\psi_{s}\left(R_{p}\right)$ is purely imaginary. Consequently, the Fourier transform of $\psi_{s}\left(R_{p}\right)$, $\Psi_{s}\left(Q_{p}\right)$ is conjugate antisymmetric.^13^

(14) $$\Psi_{s}\left(Q_{p}\right)=-\Psi_{s}^{*}\left(-Q_{p}\right).$$

Therefore, the second and third items in $G(K_{f},Q_{p})$ can be written as:

(15) $$G\left(K_{f},Q_{p}\right)={\left|A\left(K_{f}\right)\right|}^{2}\delta \left(Q_{p}\right)+\Gamma \left(K_{f},Q_{p}\right)\Psi_{s}\left(Q_{p}\right),$$

where a $\Gamma (K_{f},Q_{p})$ is introduced as in^27^:

(16) $$\begin{array}{ccc} \Gamma \left(K_{f},Q_{p}\right) & = & A^{*}\left(K_{f}\right)A\left(K_{f}-Q_{p}\right)\\ & & -\;\;A\left(K_{f}\right)A^{*}\left(K_{f}+Q_{p}\right). \end{array}$$

As for $G(K_{f},Q_{p})$ defined in Equation (9), $Q_{p}$ in Equation (16) must satisfy the condition $|Q_{p}|\leq 2\alpha$ for $\Gamma (K_{f},Q_{p})$ to be nonzero.

Taking SBm‐COMx, that is, $COM_{x}^{f}\left(R_{p}\right)$, as an example, the Fourier transform of $COM_{x}^{f}\left(R_{p}\right)$ can be represented using $\Gamma (K_{f},Q_{p})$ as follows:

(17) $$\begin{array}{ccc} F\left\{\mathrm{CO}M_{x}^{f}\left(R_{p}\right)\right\} & = & \mathrm{co}m_{x}^{f}\left(Q_{p}\right)\\ & = & \int K_{\mathrm{fx}}G\left(K_{f},Q_{p}\right)m\left(K_{f},Q_{p}\right)dK_{f}\\ & = & \int K_{\mathrm{fx}}\left[{\left|A\left(K_{f}\right)\right|}^{2}\delta \left(Q_{p}\right)+\Gamma \left(K_{f},Q_{p}\right)\right.\\ & & \left.\Psi_{s}\left(Q_{p}\right)\right]m\left(K_{f},Q_{p}\right)dK_{f}. \end{array}$$

If $|Q_{p}|\neq 0$, then the Dirac function $\delta (Q_{p})$ can be ignored and Equation (17) can be simplified as:

(18) $$\begin{array}{ccc} & & F\left\{\mathrm{CO}M_{x}^{f}\left(R_{p}\right)\right\}\\ & = & \int K_{\mathrm{fx}}\Gamma \left(K_{f},Q_{p}\right)\Psi_{s}\left(Q_{p}\right)m\left(K_{f},Q_{p}\right)dK_{f}\\ & = & \Psi_{s}\left(Q_{p}\right)\int K_{\mathrm{fx}}\Gamma \left(K_{f},Q_{p}\right)m\left(K_{f},Q_{p}\right)dK_{f}. \end{array}$$

Consequently, when $\psi_{s}\left(R_{p}\right)$, that is, inverse Fourier transform of $\Psi_{s}\left(Q_{p}\right)$, is considered as the source signal, the transfer function (TF) for $COM_{x}^{f}\left(R_{p}\right)$ is:

(19) $$TF_{x}^{f}\left(Q_{p}\right)=\int K_{\mathrm{fx}}\Gamma \left(K_{f},Q_{p}\right)m\left(K_{f},Q_{p}\right)dK_{f}.$$

Following the same analysis for $COM_{y}^{f}\left(R_{p}\right)$, the transfer function is in the same format but in the $K_{fy}$ direction given by;

(20) $$TF_{y}^{f}\left(Q_{p}\right)=\int K_{\mathrm{fy}}\Gamma \left(K_{f},Q_{p}\right)m\left(K_{f},Q_{p}\right)dK_{f}.$$

In Equations (19) and (20), as $\Gamma (K_{f},Q_{p})=0$ when $|Q_{p}|>2\alpha$, the highest frequency transferable for SBm‐COMx/y is 2α.

Based on Equation (13), the Fourier transform of SBm‐iCOM can be written as:

(21) $$\begin{array}{ccc} F\left\{iCOM^{f}\left(R_{p}\right)\right\} & = & \frac{Q_{p}\cdot \mathrm{co}m^{f}\left(Q_{p}\right)}{2\pi iQ_{p}^{2}}\\ & = & \frac{Q_{px}com_{x}^{f}\left(Q_{p}\right)+Q_{py}com_{y}^{f}\left(Q_{p}\right)}{2\pi iQ_{p}^{2}}\\ & = & \Psi_{s}\left(Q_{p}\right)\cdot \frac{Q_{px}TF_{x}^{f}\left(Q_{p}\right)+Q_{py}TF_{y}^{f}\left(Q_{p}\right)}{2\pi iQ_{p}^{2}} \end{array}$$

and the transfer function of SBm‐iCOM is:

(22) $$TF_{iCOM}^{f}\left(Q_{p}\right)=\frac{1}{2\pi }\cdot \frac{Q_{px}TF_{x}^{f}\left(Q_{p}\right)+Q_{py}TF_{y}^{f}\left(Q_{p}\right)}{iQ_{p}^{2}}.$$

In Equation (22), the numerator is zero when $|Q_{p}|>2\alpha$, meaning the highest transferable frequency for SBm‐iCOM is 2α, or equivalently, the resolution is $\frac{\lambda }{2\alpha }$. This resolution is the same as that for SSB ptychography^14^ and iCOM,^4^ as both are limited to $|Q_{p}|\leq 2\alpha$.

**FIGURE 5:**
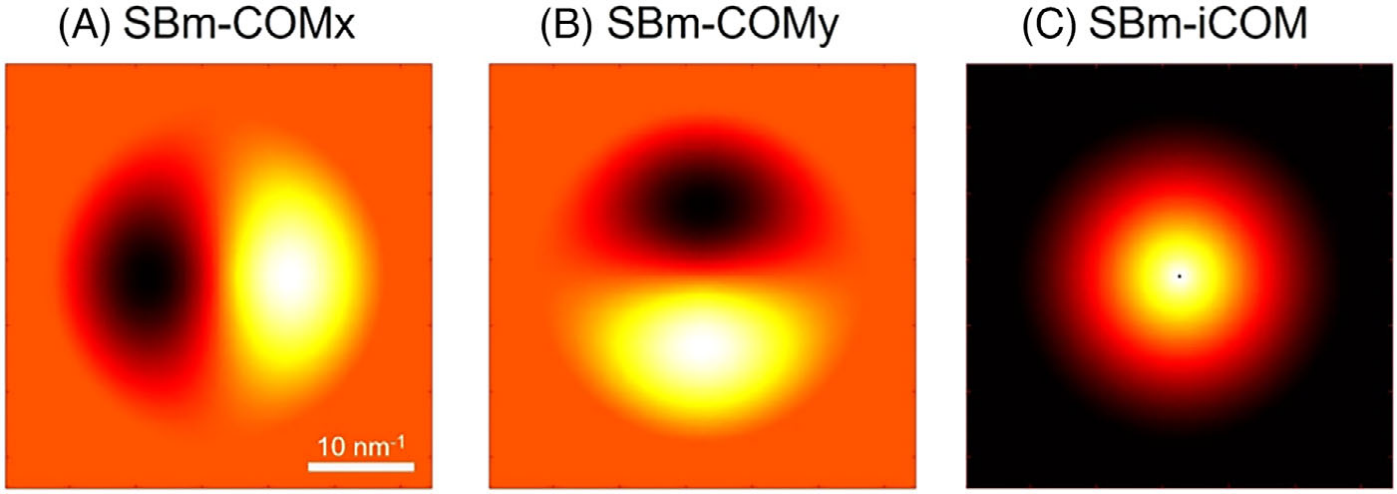
2D TF of (A) SBm‐COMx, (B) SBm‐COMy and (C) SBm‐iCOM. Simulations were calculated at 200 kV based on an aberration‐free probe with a convergence angle of 20 mrad.

Using Equations (19), (20) and (22), simulated 2D transfer functions for SBm‐COMx,y and SBm‐iCOM are shown in Figure 5.

At the high frequencies, SBm‐iCOM has the same resolution ($\frac{\lambda }{2\alpha }$) as SSB. However, the low‐frequency component (for example $|Q_{p}|<\frac{1}{2}\alpha$) differs, as SBm‐iCOM exhibits better transfer near zero frequency. This is advantageous when low‐frequency information is required. However, in cases where the sample contains noise, the low‐frequency noise may be unavoidably amplified by the transfer function as discussed later.

It is worth noting that although the transfer functions of SBm‐iCOM and SSB ptychography are different, they merely apply different modulations to the same original information $\psi_{s}\left(R_{p}\right)$ and for an ideal noise‐free condition, these can be corrected. Therefore, in principle, SBm‐iCOM does not provide more information than SSB ptychography but recovers the original signal with a different weight. This will be further discussed in the next section.

Another similar 4DSTEM imaging technique is tilt‐corrected Bright Field (tcBF) imaging.^28^ Like SBm‐iCOM, tcBF also has a resolution limit up to the frequency correspond to 2α. It treats a 4DSTEM dataset as a series of independent bright field images acquired with different beam tilts. By correcting the image shifts caused by beam tilt, tcBF achieves upsampling on the scanning screen, which is an advantage when a big field of view is needed. tcBF can also be performed under defocused conditions and has been shown to provide better contrast than EFTEM for thick biological specimens under low‐fluence conditions.^28^ The contrast transfer function of tcBF has some similarities with conventional bright field TEM, where certain spatial frequencies below 2α exhibit low information transfer due to the oscillation of its contrast transfer function. By comparison, SBm‐iCOM, being recorded in‐focus, has no zero points in its transfer function below the frequency corresponding to 2α except at zero frequency.

### 4.3 Boosted low frequencies

**FIGURE 6:**
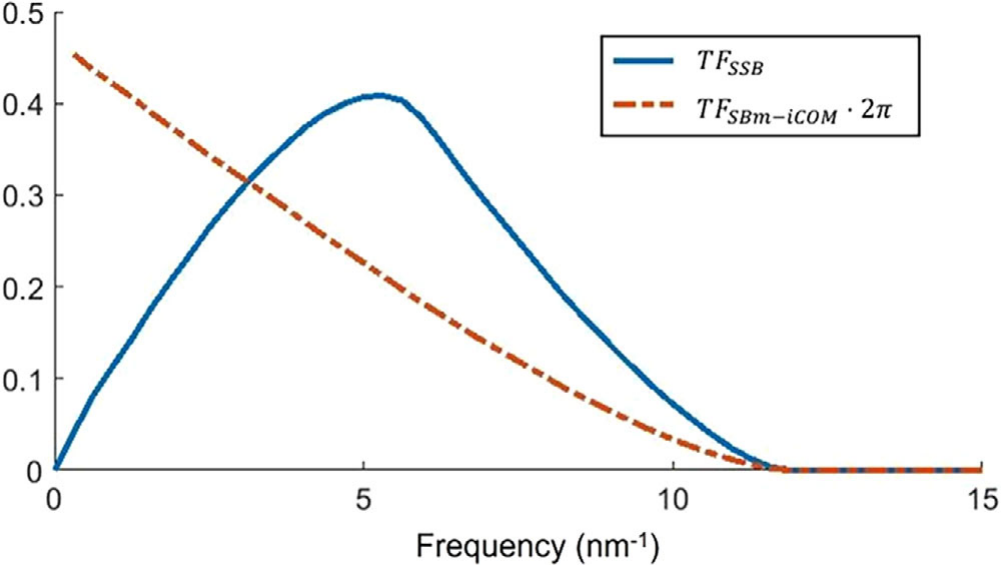
Transfer functions for SSB and SBm‐iCOM. SBm‐iCOM (scaled by 2π) shows amplified low‐frequency information transfer (below 5 nm^−1^). Data was calculated at 80 kV. Convergence angle is 25 mrad.

In addition to aberration correction and noise suppression, SBm‐iCOM reconstructs the same sample phase information within the WPOA as SSB ptychography. Their contrast transfer functions share some similarities; both tend to 0 at the frequency corresponding to 2α. However, at low frequencies, the transfer function for SBm‐iCOM, and iCOM, is higher than SSB. This arises from the $Q_{p}^{2}$ term in the denominator of Equation (22) enhancing low‐frequency transfer. One‐dimension transfer functions for SBm‐iCOM (scaled by 2π) and SSB ptychography are plotted in Figure 6 (the transfer function of SSB ptychography was calculated from Ref. (14)). This results in different reconstruction outcomes from SBm‐iCOM and SSB when the sample contains low‐frequency information. We note here that the contrast transfer function is not necessarily a good measure of information transfer because, as mentioned earlier, the regions of higher transfer function also result in higher transfer of noise. The idea of the noise‐normalised transfer function has been proposed to indicate information transfer.^29^ Here we illustrate the intrinsic effects of the algorithms without further Fourier filtering.

**FIGURE 7:**
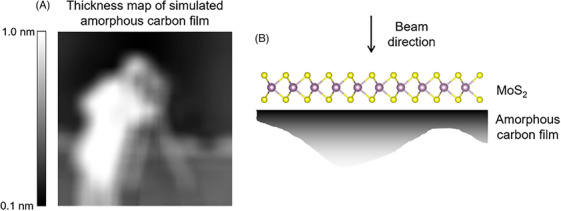
Sample used for simulation. (A) Thickness map of the nonuniform amorphous carbon film. (B) The schematic side view of the sample.

To illustrate the differences in low‐frequency transfer between SBm‐iCOM and SSB ptychography, a simulation of a perfect (defect‐free) monolayer MoS_2_ crystal on an amorphous carbon film was calculated. The amorphous carbon film was generated by randomly distributing carbon atoms within a defined volume, with the density of amorphous carbon (2.1 g/cm^3^).^30^ This model does not account for the radial distribution function of amorphous carbon since the short and medium range order is not considered. However, for the purpose of this simulation (to introduce low‐frequency noise), the effects caused by the radial distribution function can be ignored. The thickness of the carbon film was adjusted based on the thickness map shown in Figure 7A to introduce low‐frequency structural information. This nonuniform carbon film was then combined with the monolayer MoS_2_ as shown in Figure 7B.

**FIGURE 8:**
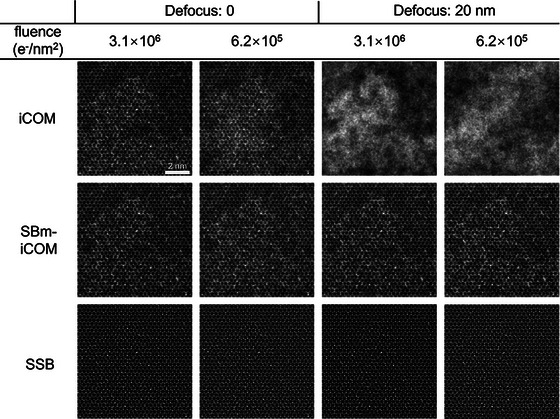
Simulated reconstructions for MoS_2_ on an amorphous carbon film. The thickness of carbon film shown in Figure 7. For both iCOM and SBm‐iCOM, the distribution of the amorphous carbon can be recognised, whereas in SSB, it is filtered out by the transfer function. For a defocus of 20 nm, SSB and SBm‐iCOM reconstruct lattice information, which is absent in the iCOM reconstruction. Acceleration voltage: 80 kV, convergence angle: 25 mrad, pixel size: 0.018 nm. Fluence used for simulation: 3.1×10^6^ e^−^/nm^2^ (corresponding to 1000 electrons per diffraction pattern) and 6.2 × 10^5^ e^−^/nm^2^ (corresponding to 200 electrons per diffraction pattern).

The simulation was based on the multislice method^31^, ^32^ using a modification of the MULTEM package.^33^ Simulation parameters are listed in Table 2 and reconstructions are shown in Figure 8 for iCOM, SBm‐iCOM and SSB ptychography.

**TABLE 2:** Simulation parameters.

Sample	Perfect monolayer MoS₂ (defect‐free)
Acceleration voltage	80 kV
Convergence angle	25 mrad
Scanning step	0.018 nm
Size of detector	64 × 64 pixels
Angular sampling on detector	1.2 mrad/pixel
Defocus	0 and 20 nm
Fluence	3.1×10^6^ and 6.2×10^5^ e^−^/nm^2^

In Figure 8, with an in‐focus probe, iCOM, SBm‐iCOM and SSB all show high contrast for the MoS_2_ lattice. However, using SSB, the carbon film background is more strongly filtered due to the suppression of low‐frequency information. For iCOM and SBm‐iCOM, the amorphous carbon background is faint but still recognisable, duo to boosted low‐frequency transfer (Figure 6). At 20 nm defocus, the aberration corrected methods, that is, SBm‐iCOM and SSB, successfully reconstruct the MoS_2_ lattice whereas iCOM fails to reconstruct the lattice structure.

**FIGURE 9:**
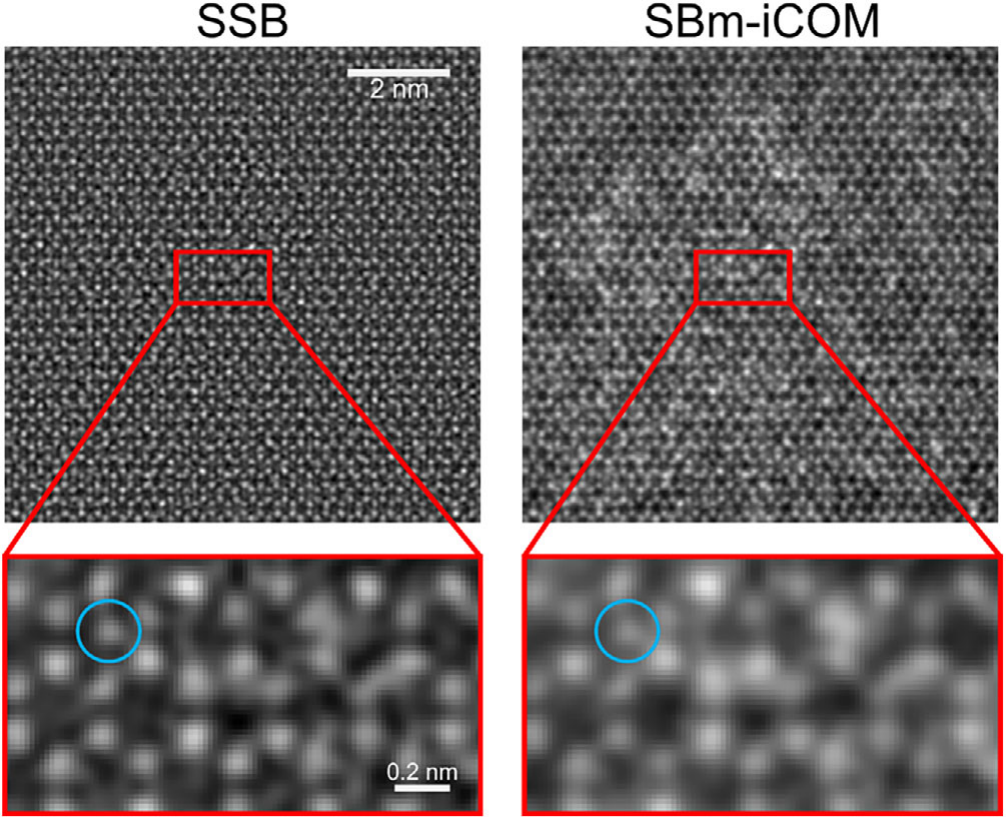
Subareas extracted from Figure 8 from simulated SSB and SBm‐iCOM reconstructions of MoS_2_ on an amorphous carbon film at 6.2 × 10^5^ e^−^/nm^2^. Blue circles indicate potentially misleading lattice positions. Acceleration voltage: 80 kV, convergence angle: 25 mrad, pixel size: 0.018 nm.

For 4DSTEM imaging, low‐frequency sensitivity can be advantageous in specific situations. In Figure 8, without prior knowledge of the carbon film, it is challenging to assess the reliability of the SSB reconstructions. When low‐frequency information, which includes details about the carbon film, is partially filtered by SSB ptychography, remaining noise could be mistaken for lattice defects, as shown in Figure 9 where in‐focus reconstructions at 6.2×10^5^ e^−^/nm^2^ (corresponding to 200 electrons per diffraction pattern) are used as examples. Blue circles in Figure 9 indicate positions that might be interpreted as additional lattice atoms.

In Figure 9, blue circles indicate misleading positions where a false ‘additional atom’ could be interpreted in both SSB and SBm‐iCOM reconstructions. However, large field‐of‐view SBm‐iCOM reconstructions provide additional low‐frequency information about the possible presence of, for example, nonuniform carbon films or contamination, which is absent in the SSB reconstruction. Therefore, if the overall contrast is influenced by substrates or unknown material with low‐frequency information, such as nonuniform carbon films or contamination, the low‐frequency information provided by SBm‐iCOM is useful for assessing the reliability of the information transferred.

However, it must be emphasised that the enhancement of low‐frequency information transfer in SBm‐iCOM does not mean that more information is transmitted; but that the weight of low‐frequency information is increased in the reconstruction.

SSB ptychography can also achieve the same low‐frequency transfer by redistributing or compensating for the intensity of different frequencies in the reconstruction. To achieve this, $\Psi_{SSB}\left(Q_{p}\right)$, the Fourier transform of a SSB reconstruction $\psi_{SSB}$, can be processed as in Equation (23):

(23) $$\begin{array}{c} \Psi_{SSB}^{\prime }\left(Q_{p}\right)=\Psi_{SSB}\left(Q_{p}\right)\cdot \frac{TF_{iCOM}^{f}\left(Q_{p}\right)}{TF_{SSB}\left(Q_{p}\right)}\\ \mathrm{where}\;\left|Q_{p}\right|>0. \end{array}$$

In Equation (23), $\Psi_{SSB}^{\prime }\left(Q_{p}\right)$ represents $\Psi_{SSB}\left(Q_{p}\right)$, modified using a frequency weight redistribution factor $\frac{TF_{iCOM}^{f}\left(Q_{p}\right)}{TF_{SSB}\left(Q_{p}\right)}$. The numerator of the factor is taken from Equation (22), while the denominator corresponds to the TF of SSB calculated as in Ref. (14). The processed SSB reconstruction can be obtained from an inverse Fourier transform to give $\Psi_{SSB}^{\prime }\left(Q_{p}\right)$. This process is similar to contrast transfer function normalisation in ptychography based on the noise distribution.^14^

**FIGURE 10:**
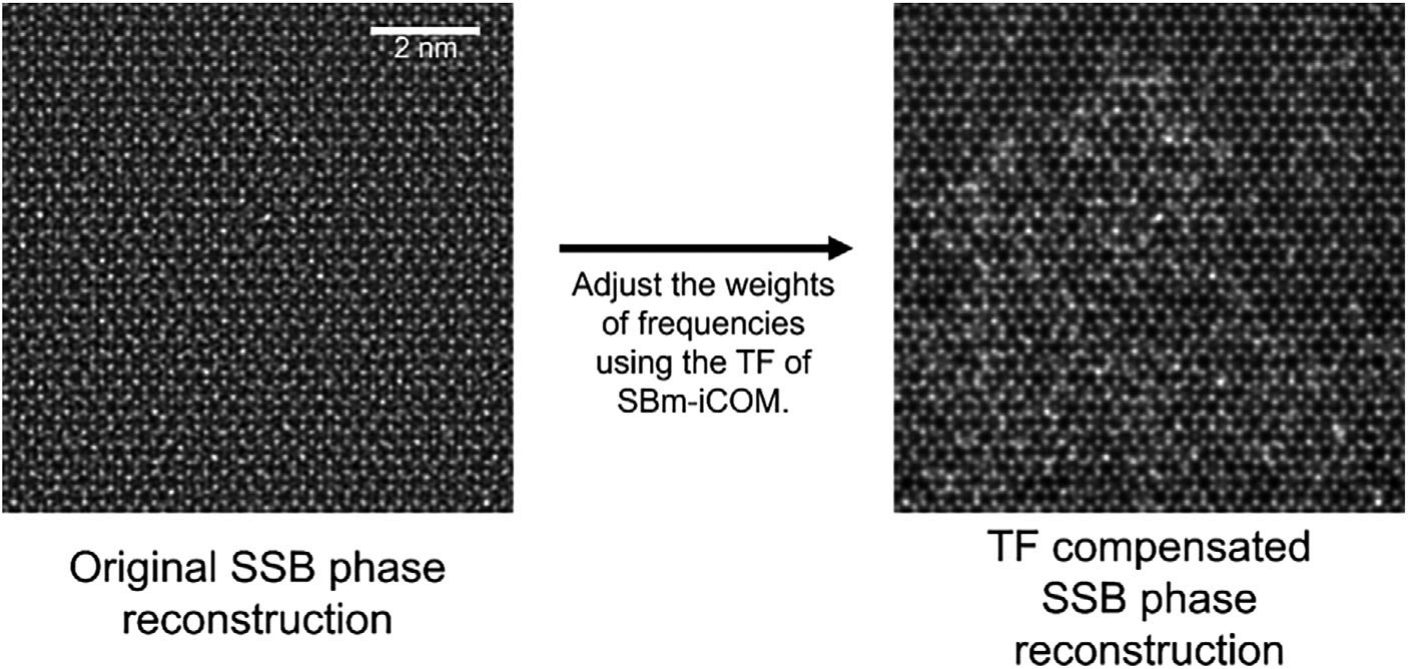
Simulated transfer function compensated SSB phase reconstruction. Sample and conditions are the same as used in Table 2 except an infinite fluence is used. The original SSB phase reconstruction is processed as described by Equation (23). The compensated SSB phase reconstruction shows enhanced low‐frequency information.

To demonstrate this, the SSB simulation results for the same sample and simulation conditions as listed in Table 2 (except for fluence, where an infinite fluence is used) are processed according to Equation (23). The original and processed SSB phase reconstructions are shown in Figure 10.

In Figure 10, the processed phase of the reconstruction (right) exhibits enhanced low‐frequency information (corresponding to the thickness map of the carbon film in Figure 9) compared to the original reconstruction (left). This is due to the low‐frequency information being enhanced by the factor $\frac{TF_{iCOM}^{f}\left(Q_{p}\right)}{TF_{SSB}\left(Q_{p}\right)}$ in Equation (23). Importantly this demonstrates that low‐frequency information is not lost in SSB reconstructions but is suppressed by the SSB transfer function and that, in theory, the SSB reconstruction contains the same information as SBm‐iCOM. The differences observed in Figure 8 between the SSB and SBm‐iCOM reconstructions therefore arise from their distinct transfer functions, rather than from fundamental differences in the information reconstructed.

In conclusion, the reconstruction results of SBm‐iCOM and SSB ptychography do not differ in terms of information transfer. However, SBm‐iCOM, compared to SSB ptychography, provides intrinsic low‐frequency enhancement without requiring additional post‐processing, such as described in Equation (23).

## 5 CONCLUSIONS

In this paper, a phase reconstruction method denoted as Side Band masked Centre‐of‐Mass (SBm‐COM), which combines SSB ptychography and Centre‐of‐Mass (COM) imaging, is discussed. In its derivation, the weak phase object approximation (WPOA) is assumed. Using SBm‐COM can compensate aberrations in a 4DSTEM dataset as is the case for SSB ptychography. However, it is not limited to an in‐focus condition as is required for traditional COM methods. For radiation sensitive samples this is an advantage since total electron fluence at the sample can be reduced by reducing the time for fine‐tuning the defocus before data collection.

An experimental dataset is shown to illustrate that SBm‐iCOM can reconstruct an atomic resolution lattice with residual defocus present in the probe. In addition, because of the SSB‐like masking process in the SBm‐iCOM method, high‐frequency noise is significantly reduced compared to traditional COM methods.

Finally, simulations are used to illustrate the frequency contrast transfer functions for SSB ptychography and SBm‐iCOM. Theoretically, SBm‐iCOM shows low‐frequency enhancement compared to SSB ptychography, although fundamentally both methods transfer the same information. For the simulations shown, a carbon film with a low‐frequency thickness variation used as a substrate was shown to give misleading results for both SSB ptychography and SBm‐iCOM. However, SBm‐iCOM provides additional low‐frequency information, although SSB with a modified TF can achieve similar reconstructions. In conclusion, SBm‐COM methods (SBm‐COMx/y and SBm‐iCOM) have the advantage of aberration‐correction and high‐frequency noise filtering as for SSB ptychography and low‐frequency transfer as for traditional COM.
